# Assessment of hydration status of elite young male soccer players with different methods and new approach method of substitute urine strip

**DOI:** 10.1186/s12970-016-0145-8

**Published:** 2016-09-02

**Authors:** Nesli Ersoy, Gulgun Ersoy, Mehmet Kutlu

**Affiliations:** 1 Department of Nutrition and Dietetics, Faculty of Health Sciences, Hacettepe University, Ankara, Turkey; 2 Department of Nutrition and Dietetics, School of Health, Turgut Ozal University, Ankara, Turkey; 3 Department of Physical Education and Sport Teaching, Faculty of Education, Kırıkkale University, Kırıkkale, Turkey

**Keywords:** Assessment of hydration status, Elite young soccer players, Dehydration, Urine strip

## Abstract

**Background:**

The purpose of the study is to determine and compare the hydration status with different methods and determine fluid intake, dehydration percentages and sweat rate of 26 young male soccer players (15 ± 1.2 years) before an important competition. More specifically, the study aims at validating the urine strip and advising the players to use it as an easy and practical method.

**Methods:**

Measurements of urine analysis were taken from the urine sample of the participants before breakfast and conducted for 3 consecutive days before the competition. Hydration status was assessed through analysis of urine color, urine specific gravity (USG) (laboratory, strip, refractometry), and osmolality. The players’ dehydration percentages and sweat ratio were calculated.

**Results:**

The average values for all samples were 3 ± 1 for color, and 1.021 ± 4 g/cm^3^ for USG (laboratory), and 1.021 ± 3 g/cm^3^ for USG (strip), and 1.021 ± 4 for USG (refractometry), and 903 ± 133 mOsm/kg for osmolality. USG (strip) was highly correlated with USG (laboratory), USG (refractometry) (*r* = 0.8; *P* < 0.01) and osmolality (*r* = 0.7; *P* < 0.01), and moderately correlated with urine color (*r* = 0.4; *P* < 0.05). The mean dehydration percentage and sweat rate of the soccer players were observed as 0.5 % and 582.3 ± 232.0 mL/h, respectively.

**Conclusion:**

We found that youth soccer players are under a slight risk of dehydration under moderate weather conditions. As indicated by the research results, determination of hydration status of athletes must be taken into account more carefully under moderate and hot weather conditions. In addition, hydration methods were compatible with one another as measured in this study.

## Background

Soccer is a team sport which involves physical performance components such as agility, speed, force, strength and endurance, and players need to have personal fluid consumption strategies [1]. Nutrition and hydration planning of adolescent soccer players, on one hand, is very important in terms of positive impact on performance parameters and growth and development, and it decreases the risk of injury on the other hand [2]. The studies have shown that dehydration causes negative impact on all physiological systems and hence the performance [3–6]. As a consequence of dehydration on muscular strength, %5 decrements on performance may be seen [7].

Over the past several years, more information and extensive series of reviews on all aspects of dehydration on specific aspects of team sports as soccer has begun to emerge, and recently been published [3, 8, 9]. It is mostly evident from these reports that there is plentiful research data relating to the hydration practices of adult soccer players, but information and research on young male soccer players is less easy to find. Owing to shortage of general information, hydration guidelines are developed for adult players [10, 11]. This is sometimes appropriate, but for some reasons this may not always be so. Some physiological and metabolic demands of young people have some differences than those of adults. Because of the fact that growth and development continue, hydration requirements of adolescent soccer players are higher/different [12–14].

Because of the importance of the determination of hydration status, it’s necessary to determine the hydration status of the athletes in a fast and confidential way. Plasma osmolality is a gold standard but it’s an expensive and impractical method and for this reason we chose urine analysis as it’s a practical, fast and less expensive method. The purpose of this descriptive study is to make a comprehensive assessment, through different methods, of hydration status (urine parameters, dehydration percentage and sweat rate) of developing young soccer players during a camp season before an important competition. More specifically, we intended to test the validity of urine strip that was not utilized in previous studies; and to advise the young soccer players to use it for finding correlation with other methods. Urine specific gravity (USG) is shown as a sensitive and valid measurement method while determining hydration status, but USG determination with urine strip is not conducted in any research.

## Methods

### Participants

Twenty-six males (ages; 15 ± 1.2 years), who volunteered to participate in this study, are playing in a Turkish youth league team (Ankaragucu youth team). All subjects were healthy, eating a normal diet and were not taking any medications that could affect their urine sample or thermoregulatory response. The study was approved by the Clinical Research Ethical Advisory Committee No. 1 in Ankara (with the resolution dated 11.11.2009 and numbered 2009/11-107), and all subjects gave their written informed consent to participation.

### Experimental design

Data were collected before an important competitive soccer match played in good weather conditions (in November) in such a way not to induce dehydration. All measurements (body weight change, food and drink consumption records and urine analysis) were taken during three consecutive days before the match. The players did their trainings on 2 days of the 3-day study period. On the next day, they were allowed to rest for the match on the following day. Temperatures and relative humidity during 3 days of tests and measurement for this study were 21.3 ± 2.5 °C and 45.3 ± 1.5 %, respectively. All players’ height and weight were measured. Their body weight was measured with a scale (Tanita BC 418MA, Tokyo, Japan) sensitive to 0.1 kg, and their height was measured with stadiometer (Seca 220, Germany).

Hydration status was determined with the urine collected (the first urine in the morning before the breakfast) by measuring the USG (g/cm^3^), urine color and osmolality (mOsm/kg). The urine samples were collected in 100 mL of sterile containers in such a way to fill minimum 50 mL. The researcher analyzed the samples at once. USG was measured with 3 different methods; laboratory (Iris 200 velocity device, Beckman Coulter, USA), strip (Combur^10^ Test M, Roche, USA), refractometry (Atago, Tokyo, Japan). In this study, hydration was determined especially from the USG result of the strip used for urine analysis (Density, pH, Leukocyte, Nitrite, Protein, Glucose, Ketone, Urobilinogen, Bilirubin, blood) in the biochemistry laboratory (Roche Combur^10^ Test M), and also its correlation with the other methods was examined. While determining the hydration status from the urine color, Armstrong 2000 color scale was used [7]. Urine osmolality was determined via freezing point depression of the samples measured with Osmometer-3320 (Germany).

Similarly, players’ body weight was measured before and after the trainings which went on for 2 days, calculating the amount of their fluid loss during the trainings. Sweat loss was assessed from the change in body mass after correction for the volume of fluid consumption and urine excretion. They gave urine samples before breakfast and afterwards (when their bladder was empty), and their body weight was measured with a scale (Tanita BC 418MA, Tokyo, Japan) sensitive to 0.1 kg with their dry training clothes, shorts and t-shirts on. During the exercise, the urine voided and the fluid they consumed was recorded. All subjects had unrestricted access to water, and all water bottles were individually numbered and players only drank from bottles assigned to them. All water bottles were weighed on electronic scales (CWT, Dikomsan, Istanbul, TR), measuring to the nearest 1 g, before and at the end of the exercise to assess the volume consumed by each player. The body weight of the players was measured with the same scale after they took shower and dried themselves (while the bladder was empty), and the amount of the fluid lost during the exercise, the sweat ratio and the dehydration rates were calculated. In these calculations and sweat loss calculations, mass changes due to metabolism and respiratory water loss were considered negligible. Players watched carefully during the training and they consumed the fluid in the bottles.

$$ Sweat\  ratio:\left(\mathrm{A} + \mathrm{B}\right)\div \mathrm{C} $$

A = Pre-exercise body weight (kg) – Post-exercise body weight (kg),

B = Fluid Consumed during Exercise (mL) – urine excretion amount during exercise (mL),

C = Weight

$$ Dehydration\  percentage:\left(\mathrm{P}\mathrm{r}\mathrm{e}\hbox{-} \mathrm{exercise}\ \mathrm{body}\ \mathrm{weight}\ \left(\mathrm{kg}\right)\ \hbox{--} \mathrm{Post}\hbox{-} \mathrm{exercise}\ \mathrm{body}\ \mathrm{weight}\ \left(\mathrm{kg}\right)\times 100\right)/\mathrm{P}\mathrm{r}\mathrm{e}\hbox{-} \mathrm{exercise}\ \mathrm{body}\ \mathrm{weight}\ \left(\mathrm{kg}\right). $$

### Statistical analyses

Data were analyzed using SPSS 15 software. Descriptive statistics on personal characteristics, and hydration variables was calculated. Means and average values and standard deviation values of food consumption, nutritional support products and fluid intake were also determined for all subjects. Relationships between parameters were investigated by Spearman’s correlation test. The level of statistical significance was set as *P < 0.05*.

## Results

### Participants

Physical characteristics of the players were: age 15 ± 1.2 years, height 175.2 ± 6.8 cm, and weight 67.3 ± 5.9 kg. The soccer players were playing soccer for an average period of 7 years; and they were doing training for 6 ± 0.6 days (110 ± 14.6 min a day) in a week.

### Hydration status

Fluid consumption, body weight monitoring and urine parameters were measured in order to assess hydration status during soccer training. It was observed that the drinking water and fluid were obtained from the food and beverages for a period of 3 days. Average daily fluid consumption of the players during 2 days of training was found to be 2780 ± 567 mL, while their water consumption during the trainings was found to be 908.6 ± 332.7 mL.

Dehydration percentages and sweat ratio calculated according to the changes in the body weights of the soccer players on 2 training days are shown in Table 1. The dehydration percentage of the soccer players on the first day, second day and the mean value of the 2 days were observed as 0.3, 0.8 and 0.5 %, respectively.

**Table 1:** Dehydration percentages and sweat ratio according to the body weights of the soccer players before and after the exercise

Days	Dehydration %	Sweat ratio (mL/h)	*P*
	$\overline{x}\pm \mathrm{S}\mathrm{D}$	$\overline{x}\pm \mathrm{S}\mathrm{D}$	
1^st^ day	0.3 ± 0.6	668.5 ± 268.0	0.011
2^nd^ day	0.8 ± 1.2	496.2 ± 231.6	0.080
Mean	0.5 ± 0.7	582.3 ± 232.0	

The average and standard deviation values of 3-day urine analysis results of the soccer players are shown in Table 2.

**Table 2:** The 3-day urine analysis results of the soccer players as hydration determination methods

Urine parameters	$\overline{x}\pm \mathrm{S}\mathrm{D}$	Min-Max
Colour	3 ± 1	2–4
Specific gravity (laboratory-g/cm^3^)	1.021 ± 4	1.014–1.026
Specific gravity (strip-g/cm^3^)	1.021 ± 3	1.015–1.025
Specific gravity (refractometry)	1.021 ± 4	1.015–1.026
Osmolality (mOsm/kg)	903 ± 133	656–1171

### Validity of urine strip

Correlation coefficients on the urine analysis indicators used in order to determine the validity of urine USG measurement and osmolality for determination of hydration status of the soccer players are shown in Table 3. Urine color, USG (laboratory, strip and refractometry) and osmolality analyses were made in order to determine the hydration status of the soccer players; and it was observed that statistically all the urine measurement methods were highly correlated among themselves (*P* < 0.05). USG (strip) was also highly correlated with USG (laboratory), USG (refractometry) (*r* = 0.8; *P* < 0.01) and osmolality (*r* = 0.7; *P* < 0.01), but only moderately correlated with urine color (*r* = 0.4;*P* < 0.05).

**Table 3:** Correlation coefficients between variables for all test of urine samples *(n* = 26)

Parameters		Colour	Specific gravity (laboratory)	Specific gravity (strip)	Specific gravity (refractometry)	Osmolality
Colour		1.000	0.626**	0.441*	0.663**	0.598**
	*P*	.	0.001	0.024	0.000	0.001
Specific gravity (laboratory-g/cm^3^)		0.626**	1.000	0.807**	0.912**	0.921**
	*P*	0.001	.	0.000	0.000	0.00
Specific gravity (strip-g/cm^3^)		0.441*	0.807**	1.000	0.750**	0.709**
	*P*	0.024	0.000	.	0.000	0.000
Specific gravity (refractometry)		0.663**	0.912**	0.750**	1.000	0.886**
	*P*	0.000	0.000	0.000	.	0.000
Osmolality (mOsm/kg)		0.598**	0.921**	0.709**	0.886**	1.000
	*P*	0.001	0.000	0.000	0.000	.

**P < 0.01

*P < 0.05

## Discussion

Dehydration not only has negative effects on sports performance but also leads to health problems which maypotentially put life at risk [15]. As a result of this study attempting to find out hydration status of adolescent soccer players, it was found that the athletes are under the risk of dehydration, though with a low level, in terms of fluid consumption and hydration. The study found that the athletes’ hydration levels were not highly affected due to the fact that the temperature and humidity were not at levels which trigger dehydration, but they still needed hydration support. Different analysis including urine color, urine density (strip, device and refractometry) and osmolality methods were utilized in finding the hydration status, and it was discovered that urine density stripes provide a safe method with a practical use in the field, making it possible to substitute it for other methods. Due to the limited number of studies in the world and our country on hydration status of adolescent athletes, particularly that of soccer players, it is thought that the present study will contribute to the science and related sports branch.

The most important finding of this study is the significant positive correlation of urine hydration methods. There are some studies assessing the validity of urine hydration methods [16–18]. Popowski et al. [17] reported that plasma osmolality, body weight change, urine osmolality and USG were determined as sensitive analysis methods, which may substitute for one another. In another study aiming to determine hydration status of the taekwondo players and comparison of methods, it was found that there was a strong correlation among urine osmolality, urine electrical conductivity, urine color and USG [16]. We also found a significant correlation among urine color, USG (laboratory, strip and refractometry) and osmolality analyses. Especially, USG (strip) was also highly correlated with USG (laboratory) (*r* = 0.81;*P* < 0.01), USG (refractometry) (*r* = 0.75; *P* < 0.01) and osmolality (*r* = 0.71;*P* < 0.01), but only moderately correlated with urine color (*r* = 0.44;*P* < 0.05) [16, 19, 20]. It is thought that the reason why urine color method had low correlation is because urine color hydration analysis has more error margin compared to other methods since it depends on the outcome of observation and resulting interpretation. It was concluded as a result of the present study that urine strip gave similar results with urine osmolality, USG (laboratory and refractometry) and color. Urine color and USG are reliable methods, which may be used in situations where laboratory measurements and measurement equipment are not available for use [16, 21]. Similar to this study, we can also recommended to young athletes urine strip (USG analysis) method as a practical and cheap hydration method, which may be used in the field.

Another important finding of this study is that low level dehydration occured during exercise in slightly moderate weather conditions, and low level of dehydration is even crucial for adolescence soccer players because as their growth and development continues, hydration requirements of adolescent soccer players are higher/different than adult soccer players [12–14]. Dehydration not only leads to negative impacts on sports performance but also causes health problems, which may endanger life [22–24]. Moderate level of dehydration occurs during training due to heavy metabolic activity and hot environment conditions [25]. As an objective noninvasive measurement, it is possible to calculate sweat ratio and dehydration percentages of the athletes through monitoring their body weight before and after training [5]. In a study conducted on a Kuwaiti soccer team, it was observed as a result of monitoring the body weight changes before and after training that the soccer players lost 3.1 ± 1.4 L fluid and completed the training in a dehydrated state (urine specific gravity = 1.026 ± 0.002 g/cm^3^) [26]. In a similar study, body weights of 76 adolesence soccer players, whose mean age is 15.9 ± 0.8 years, were monitored before and after the training and it was determined at the end of the training that they were dehydrated by 0.7 ± 0.7 % [27]. It was observed in a study conducted on a UK Premier League soccer team that the soccer players consumed 971 ± 303 mL fluid and lost 2033 ± 413 mL of sweat during training and that they lost 1.37 ± 0.54 % of their body weight [8]. In another study conducted it was determined that the sweat ratio of the soccer players varied between 985 and 1209 mL/h [28], however in this present study, the sweat ratio was observed as 582.3 ± 232.0 mL/h. In this present study, it was observed that the fluid consumption of the soccer players during training was 908.6 ± 332.7 mL, and the fluid amount lost through sweat was 873.5 ± 348 mL. 12 of the participants did not replace their body water with fluid consumption and they finished the training in a dehydrated condition although their average dehydration percentage was as low as 0.5, and that the dehydration percentages were not at a level to have negative impact on their soccer performance. According to the results of the study, although it was found that the athletes recovered the fluid they lost during the training, it is estimated that they normally consumed much less water during the training. In the stage before the study where the athletes were informed about the study, and where they signed consent forms, they developed awareness about the study. It is believed that due to their young age, they paid attention to consuming water because of the concern that the study would be an assessment of their performance, and that is why they consumed more water.

In this study it’s determined that urine methods are consistent with each other. In future studies, urine strip’s consistency with plasma osmolality should be assessed.

## Conclusion

Hydration status of all athletes should be determined at regular intervals. There is not a single gold standard in determination of the hydration status including plasma and urine osmolality, and it should not be expected that all measurement techniques exactly give the same result. In this present study, it has been determined that urine color and USG methods and especially strip method are reliable and valid analysis methods, which may be used in the field. Measuring the body weight of the athletes in 3 consecutive days or monitoring their body weight before and after training are practical methods used in the field (Shirreffs, [10]; Shirreffs, Sawka & Stone, [11]). In addition to monitoring the body weight daily while the bladder is empty and the athletes are hungry, usage of urine analyses (color, USG and osmolality) are the most effective methods in determination and monitoring of the hydration status. The body weight measurements taken every morning should be kept within the range of 2 % [10, 11]. On those days, the urine color should be monitored, and attention must be paid that the urine color is light yellow [7]. Similarly, the body weight and the fluid consumption of the players should be monitored before and after training, and the fluid amount lost during training can be determined.

The coaches, team workers, players and their families are important parts of the hydration education and protocol. The first target in the hydration training to be given to the players by the dieticians is the fluid selection and hydration follow-up (body weight change and urine color). Effective educational interventions are still necessary to prevent dehydration during matches.

It should be useful to repeat this study in hot (summer months) and cold (winter months) weather conditions in order to determine the hydration status of the young soccer players.

## Abbreviation

USG: Urine specific gravity
